# Supplementary material for: Tryptophan fuels MYC-dependent liver tumorigenesis through indole 3-pyruvate synthesis
Source: Nat Commun. 2024 May 20;15:4266. doi: 10.1038/s41467-024-47868-3 (PMC11106337; doi:10.1038/s41467-024-47868-3)
Supplement: Supplementary file 1 — Supplemental information [file 41467_2024_47868_MOESM1_ESM.pdf]

Supplementary Information

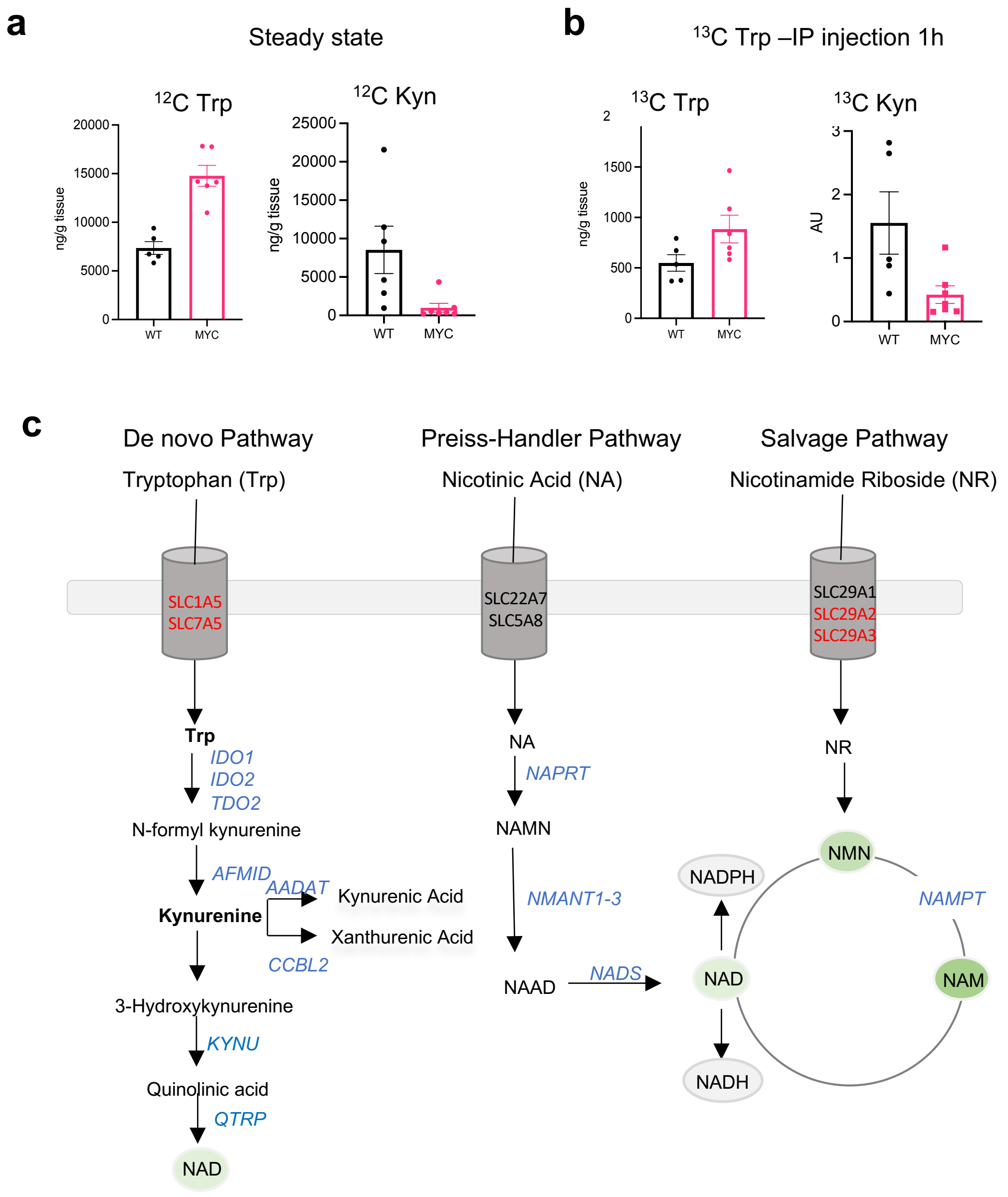
a
Steady state
^{12}C Trp
ng/g tissue
20000
15000
10000
5000
0
WT
MYC
^{12}C Kyn
ng/g tissue
25000
20000
15000
10000
5000
0
WT
MYC
b
^{13}C Trp –IP injection 1h
^{13}C Trp
2
ng/g tissue
1500
1000
500
0
WT
MYC
^{13}C Kyn
AU
3
2
1
0
WT
MYC
c
De novo Pathway
Tryptophan (Trp)
Preiss-Handler Pathway
Nicotinic Acid (NA)
Salvage Pathway
Nicotinamide Riboside (NR)
SLC1A5
SLC7A5
SLC22A7
SLC5A8
SLC29A1
SLC29A2
SLC29A3
Trp
IDO1
IDO2
TDO2
N-formyl kynurenine
AFMID
Kynurenine
AADAT
Kynurenic Acid
Xanthurenic Acid
CCBL2
3-Hydroxykynurenine
KYNU
Quinolinic acid
QTRP
NAD
NA
NAPRT
NAMN
NMANT1-3
NAAD
NADS
NR
NMN
NADPH
NAMPT
NAD
NAM
NADH

Figure S1

Supplemental Fig. 1: MYC-driven liver tumors exhibit an increase in Trp uptake.

(**a**) Quantification of Trp and Kyn in WT and MYC-ON livers.

(**b**) Quantification of ^{13}C Trp and ^{13}C Kyn in WT and MYC-ON livers.

(**c**) Schematic of the NAD metabolism pathway depicting the different routes of production of NAD^+. Upregulated genes are red, downregulated genes are blue. Plots show mean +/− SD and *P*-value was calculated by *t*-test.

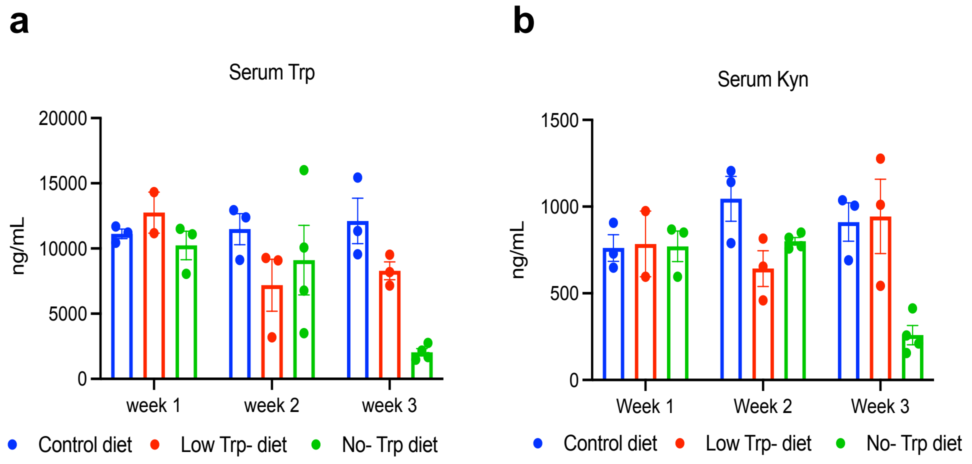
a
Serum Trp
ng/mL
20000
15000
10000
5000
0
week 1
week 2
week 3
b
Serum Kyn
ng/mL
1500
1000
500
0
Week 1
Week 2
Week 3
Control diet
Low Trp- diet
No- Trp diet

Figure S2

Supplemental Fig. 2: Trp starvation for 3 weeks is necessary to reduce the levels of Trp and Kyn in the serum. Quantification of Trp (**a**) and Kyn (**b**) in the liver of C57/B6 mice on the indicated diets for 1, 2, and 3 weeks.

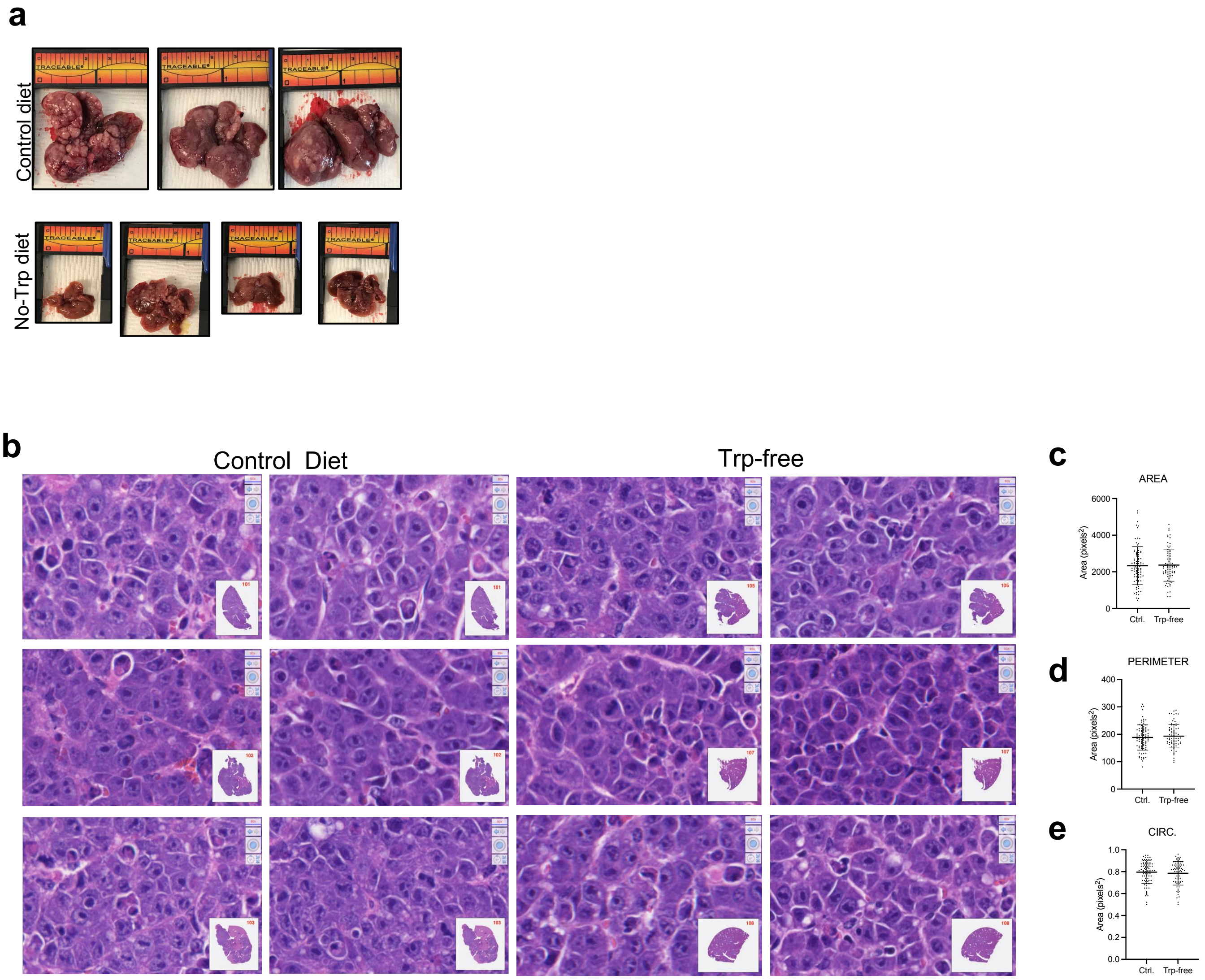
a
Control diet
No-Trp diet
b
Control Diet
Trp-free
c
AREA
Area (pixels²)
6000
4000
2000
0
Ctrl.
Trp-free
d
PERIMETER
Area (pixels²)
400
300
200
100
0
Ctrl.
Trp-free
e
CIRC.
Area (pixels²)
1.0
0.8
0.6
0.4
0.2
0.0
Ctrl.
Trp-free

Figure S3

Supplemental Fig. 3: Trp starvation prevents MYC-driven liver tumors from arising.

(a) Pictures of the livers of animals fed either the control or the No-Trp diet after 21 days.

(**b**) H & E sections obtained from MYC-ON mice that were fed either the control or No-Trp diet for 21 days. Tumor sections are shown at 80X magnification.

(**c**) Nucleolar area measurements from (A), comparing livers from mice fed either the control or No-Trp diet in the MYC-On mice. (Ctrl. N= 89, No-Trp N=75) Each dot represents one mouse. Plots show mean +/− SD and *P*-value was calculated by *t*-test.

(**d**) Nucleolar perimeter measurements from (A), comparing livers from mice fed either the control or No-Trp diet in the MYC-On mice. (Ctrl. N= 89, No-Trp N=75) Each dot represents one mouse. Plots show mean +/− SD and *P*-value was calculated by *t*-test.

(**e**) Nucleolar circularity measurements from (a), comparing livers from mice fed either the control or No-Trp diet in the MYC-On mice. (Ctrl. N= 89, No-Trp N=75) Each dot represents one mouse. Plots show mean +/− SD and *P*-value was calculated by t-test. $^*P \leq 0.05$.

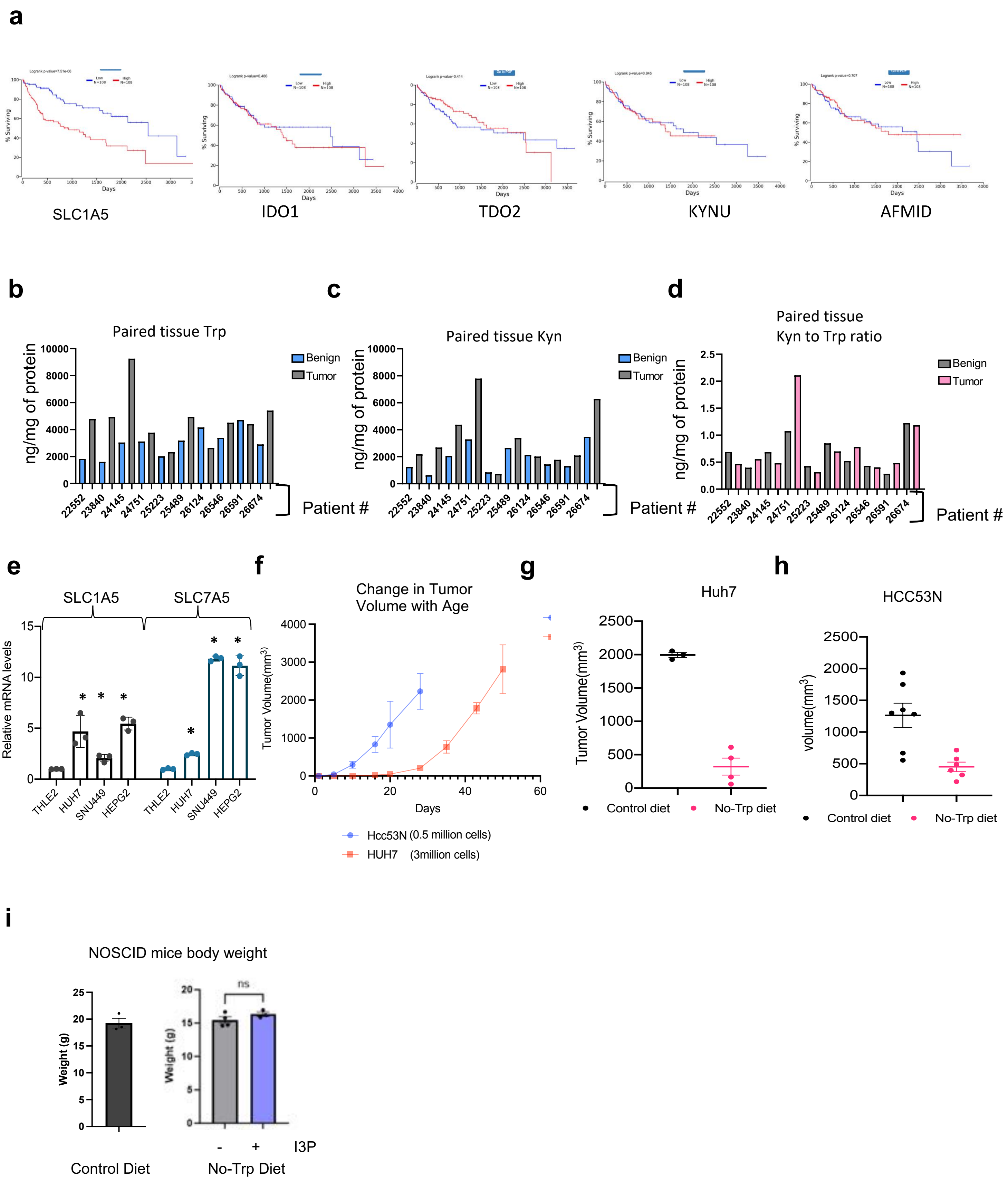

a
SLC1A5
IDO1
TDO2
KYNU
AFMID
% Surviving
Days
b
Paired tissue Trp
ng/mg of protein
Benign
Tumor
Patient #
22552
23840
24145
24751
25223
25489
26124
26546
26591
26674
c
Paired tissue Kyn
d
Paired tissue
Kyn to Trp ratio
e
SLC1A5
SLC7A5
Relative mRNA levels
THLE2
HUH7
SNU449
HEPG2
f
Change in Tumor
Volume with Age
Tumor Volume(mm3)
Days
Hcc53N (0.5 million cells)
HUH7 (3million cells)
g
Huh7
Tumor Volume(mm3)
Control diet
No-Trp diet
h
HCC53N
volume(mm3)
Control diet
No-Trp diet
i
NOSCID mice body weight
Weight (g)
ns
I3P
Control Diet
No-Trp Diet

Figure S4

Supplemental Fig. 4: Trp transporters are elevated in liver cancer cell lines and Trp starvation has a cell autonomous effect of inhibiting tumor growth *in vivo*.

(a) Kaplan-Meier curve showing the survival of patients expressing high levels (30% highest) of *SLC1A5, IDO1, TDO2, KYNU, AFMID,* and low (30% lowest) levels of SLC1A5. Data obtained from the ONCOLNC database.

(b-c) Quantification of Trp (b) and Kyn (c) in individual tissues samples determined to be benign or tumor from 10 patients diagnosed with HCC, obtained from the UTSW biorepository.

(**d**) Ratio of Kyn to Trp of the patient samples in (b-c) representing percentage of Trp converted to Kyn for each patient.

(**e**) Relative mRNA levels of Trp transporters in the used cell lines. Comparison relative to THLE2 cells. *RPS18* was used as a housekeeping gene.

(**f**) Characterization of the growth of HUH7 and HCC53N cells transplanted into NOD SCID mice.

(**g**) Tumor volume of HUH7 cells transplanted into NOD SCID mice and grown for 21 days in mice fed either the control or No-Trp diet.

(**h**) Tumor volume of HCC53N cells transplanted into NOD SCID mice and grown for 21 days in mice fed either the control or No-Trp diets.

(**i**) Weight of mice that were assigned to receive either the control or No-Trp diet for 21 days (n=4 mice). $*P \leq 0.05$. Each dot represents one mouse. Plots show mean +/− SEM and *P*-value was calculated by *t*-test.

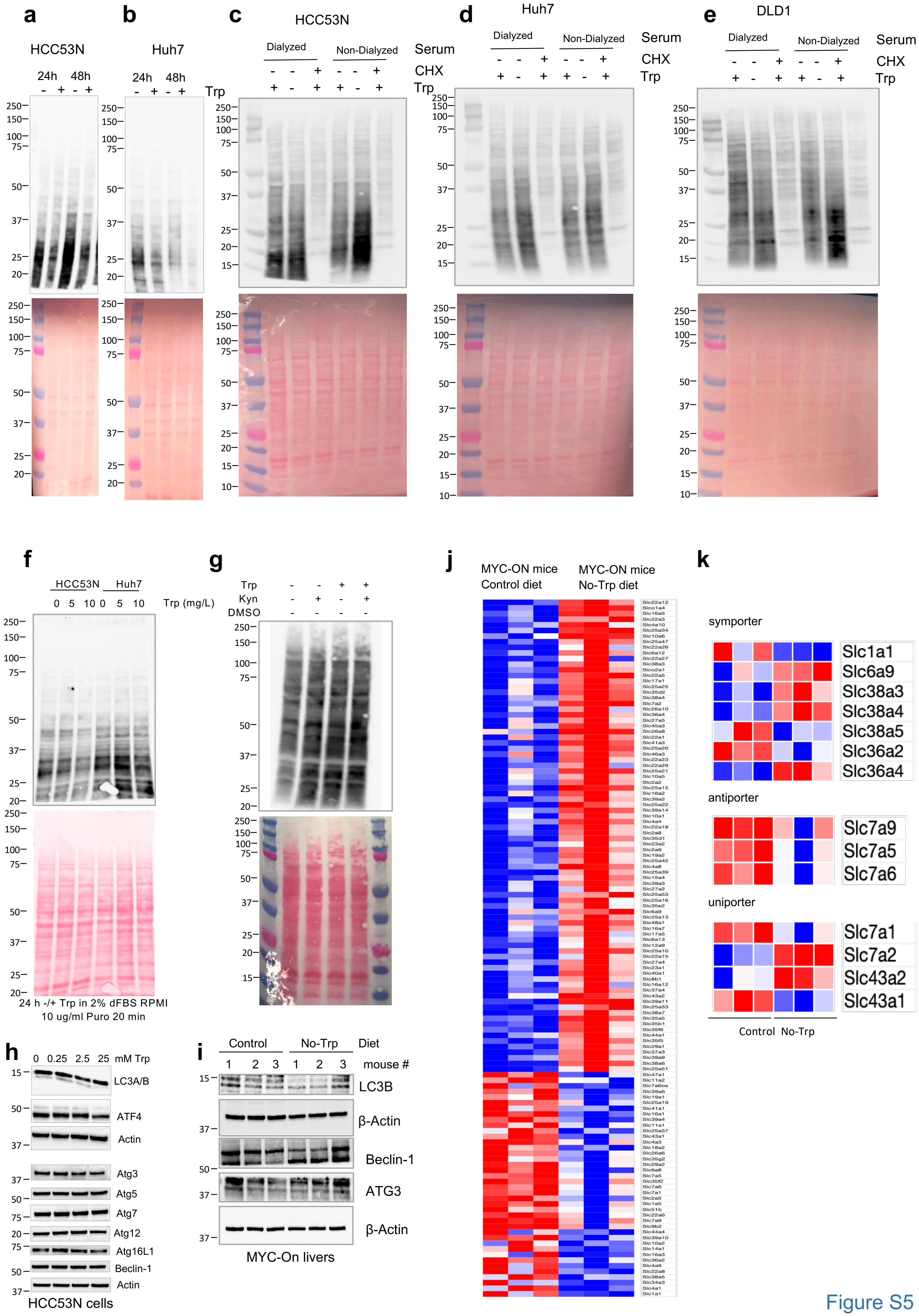
a
HCC53N
24h 48h
- + - +
b
Huh7
24h 48h
- + - +
Trp
c
HCC53N
Dialyzed
Non-Dialyzed
Serum
- - + - - +
CHX
+ - + + - +
Trp
d
Huh7
Dialyzed
Non-Dialyzed
Serum
- - + - - +
CHX
+ - + + - +
Trp
e
DLD1
Dialyzed
Non-Dialyzed
Serum
- - + - - +
CHX
+ - + + - +
Trp
250
150
100
75
50
37
25
20
15
10
f
HCC53N
Huh7
0 5 10 0 5 10
Trp (mg/L)
24 h -/+ Trp in 2% dFBS RPMI
10 ug/ml Puro 20 min
g
Trp
Kyn
DMSO
- - + +
- + - +
- - - -
h
0 0.25 2.5 25 mM Trp
LC3A/B
ATF4
Actin
Atg3
Atg5
Atg7
Atg12
Atg16L1
Beclin-1
Actin
HCC53N cells
i
Control
No-Trp
Diet
1 2 3 1 2 3
mouse #
LC3B
β-Actin
Beclin-1
ATG3
β-Actin
MYC-On livers
j
MYC-ON mice
Control diet
MYC-ON mice
No-Trp diet
k
symporter
Slc1a1
Slc6a9
Slc38a3
Slc38a4
Slc38a5
Slc36a2
Slc36a4
antiporter
Slc7a9
Slc7a5
Slc7a6
uniporter
Slc7a1
Slc7a2
Slc43a2
Slc43a1
Control
No-Trp

Figure S5

Supplemental Fig. 5: Trp starvation does not affect protein synthesis in liver cancer cells.

(a-b) Puromycylation of HCC53N (A) and HUH7 (B) cells grown for 24 or 48 h in the presence or absence of 75 μM Trp.

(c-e) Puromycylation of HCC53N (c) HUH7 (d), and DLD1 (e) cells grown for 24 h in the presence or absence of 75 μM Trp with either dialyzed or non-dialyzed serum. CHX was used at 50 mg/mL to inhibit protein synthesis.

(f) AHA click chemistry used to measure protein synthesis initiation in HCC53N and HUH7 cells grown in increasing concentrations of Trp. Experiment performed in cells incubated for 24 h with increasing amounts of Trp.

(g) Puromycylation of HUH7 cells in the presence of Trp (75 μM) or Kyn (20 μM) overnight.

(h) Western blot for the indicated proteins in HCC53N cells grown in increasing amounts of Trp for 24 h.

(i) Western blot for the indicated proteins in extracts from MYC-ON livers from mice fed either the control or No-Trp diet for 21 days.

(j) mRNA expression of SLC (s) from MYC-ON livers from mice fed either the control or No-Trp diet for 21 days.

(k) mRNA expression of amino acid transporters in MYC-ON livers from mice fed either the control or No-Trp diet for 21 days.

a

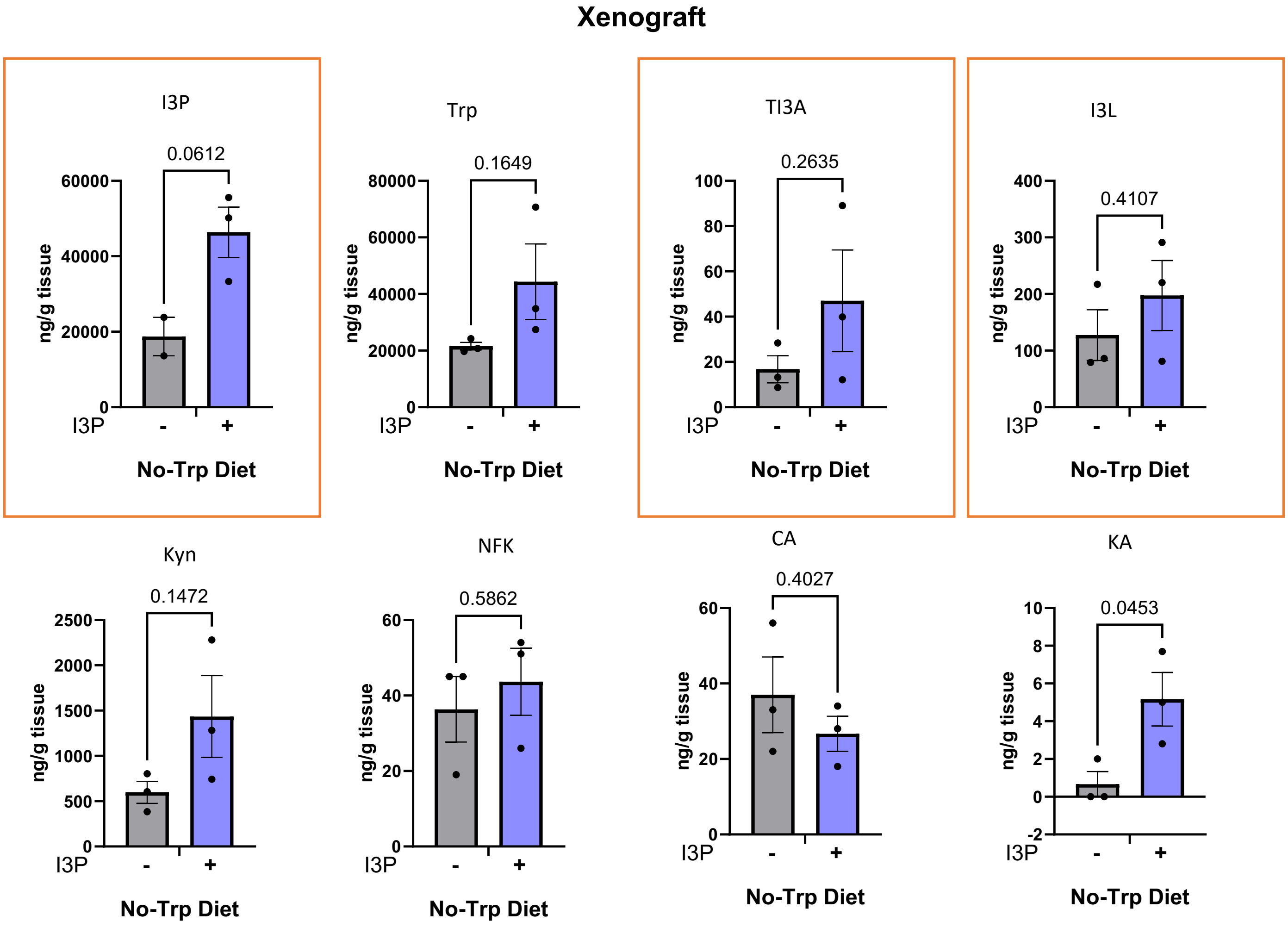
Xenograft
I3P
0.0612
ng/g tissue
60000
40000
20000
0
I3P
-
+
No-Trp Diet
Trp
0.1649
80000
60000
40000
20000
0
No-Trp Diet
TI3A
0.2635
100
80
60
40
20
0
No-Trp Diet
I3L
0.4107
400
300
200
100
0
No-Trp Diet
Kyn
0.1472
2500
2000
1500
1000
500
0
No-Trp Diet
NFK
0.5862
60
40
20
0
No-Trp Diet
CA
0.4027
60
40
20
0
No-Trp Diet
KA
0.0453
10
8
6
4
2
0
-2
No-Trp Diet

Figure S6

Supplemental Fig. 6. Alterations in Trp and its metabolites in HCC53N xenografts grown in mice fed a control or No-Trp diets in the presence or absence of I3P supplementation. Orange box marks the graph used in Figure 7(q). The measurement was performed by LC-MS/MS. Plot shows mean +/- SEM and *P*-value was calculated by unpaired *t*-test. n=3 for vehicle and n=3 for I3P.

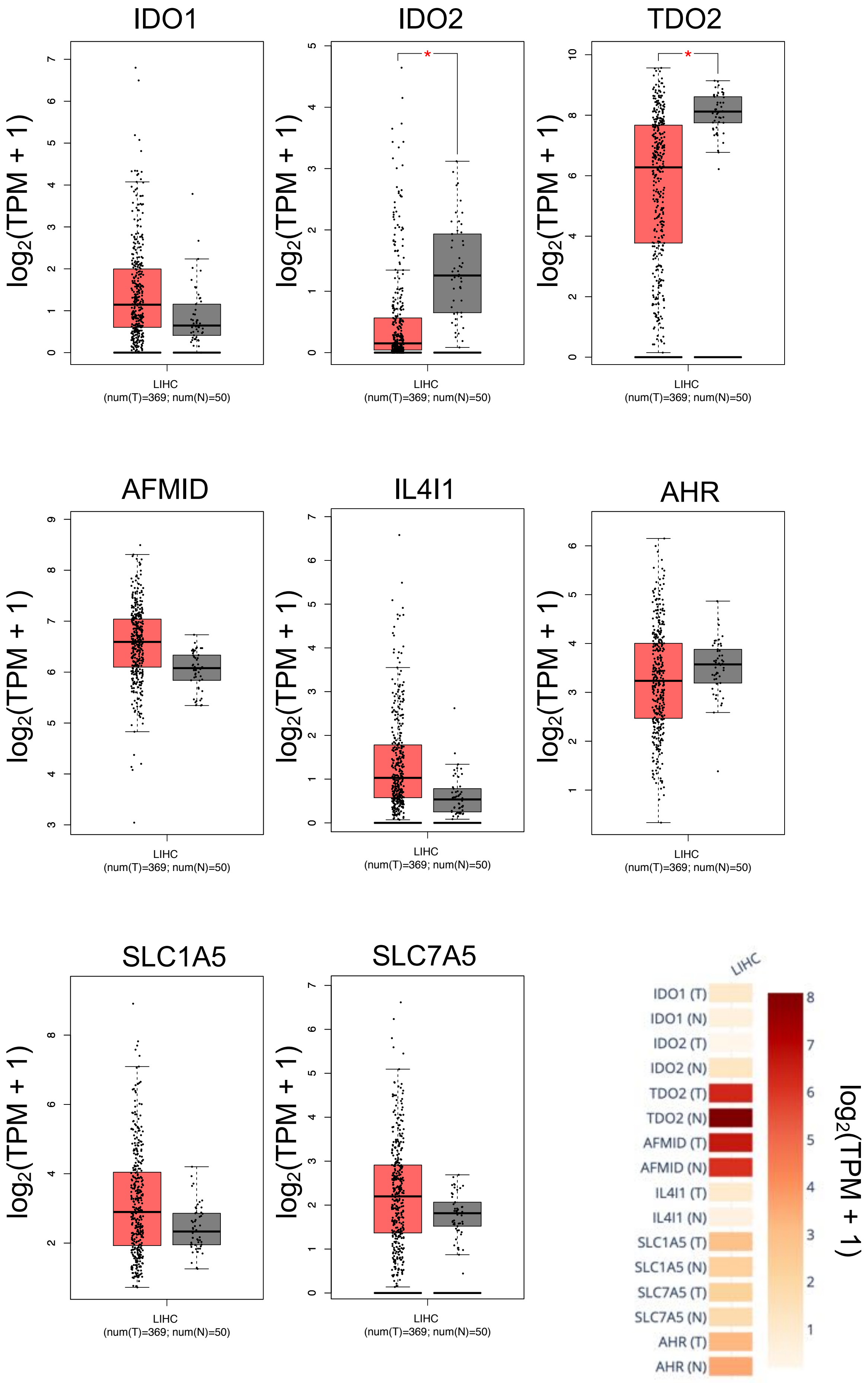
IDO1
IDO2
TDO2
AFMID
IL4I1
AHR
SLC1A5
SLC7A5
log2(TPM + 1)
LIHC
(num(T)=369; num(N)=50)
*
IDO1 (T)
IDO1 (N)
IDO2 (T)
IDO2 (N)
TDO2 (T)
TDO2 (N)
AFMID (T)
AFMID (N)
IL4I1 (T)
IL4I1 (N)
SLC1A5 (T)
SLC1A5 (N)
SLC7A5 (T)
SLC7A5 (N)
AHR (T)
AHR (N)

Figure S7

Supplemental Fig. 7: Expression of Trp-metabolizing enzymes and Trp transporters in normal livers and liver tumors in the TCGA database.

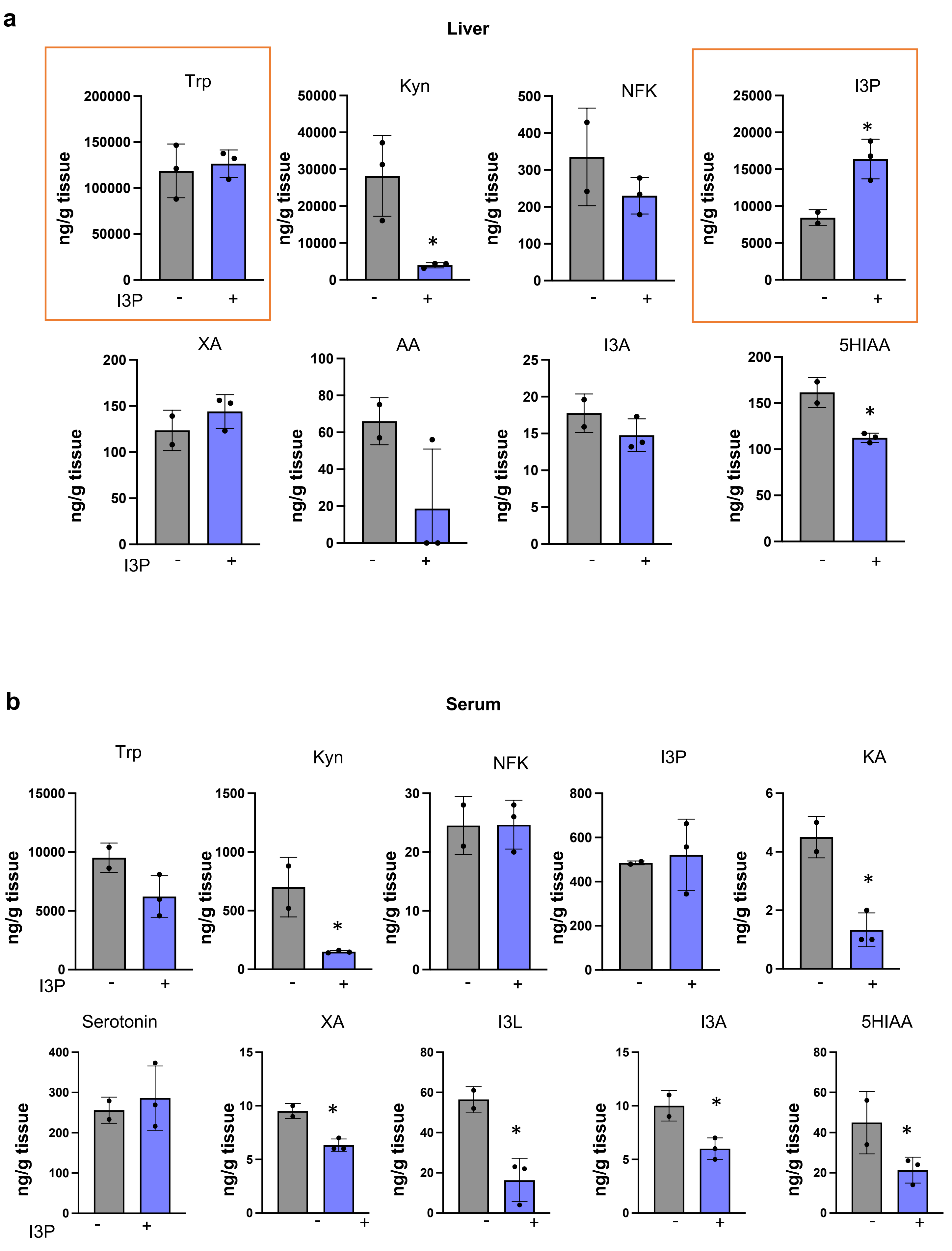
a
Liver
Trp
Kyn
NFK
I3P
ng/g tissue
200000
150000
100000
50000
0
50000
40000
30000
20000
10000
500
400
300
200
100
25000
20000
15000
10000
5000
I3P
-
+
*
XA
AA
I3A
5HIAA
b
Serum
Trp
Kyn
NFK
I3P
KA
15000
10000
5000
1500
1000
500
30
20
10
800
600
400
200
6
4
2
Serotonin
XA
I3L
I3A
5HIAA
400
300
200
100
15
10
5
80
60
40
20

Figure S8

Supplemental Fig. 8: Alterations in Trp and its metabolites in the livers and serum of MYC-ON mice fed either the control or No-Trp diet in the presence or absence of I3P supplementation.

(a) Trp and I3P levels in MYC-ON livers of animals fed the No-Trp diet supplemented with daily IP injections of either vehicle or I3P (75 μM) for 14 days. Orange boxes indicate graphs shown in Figure 8 (j).

(b) Trp and I3P levels in MYC-ON serum of animals fed the No-Trp diet supplemented with daily IP injections of either vehicle or I3P (75 μM) for 14 days.

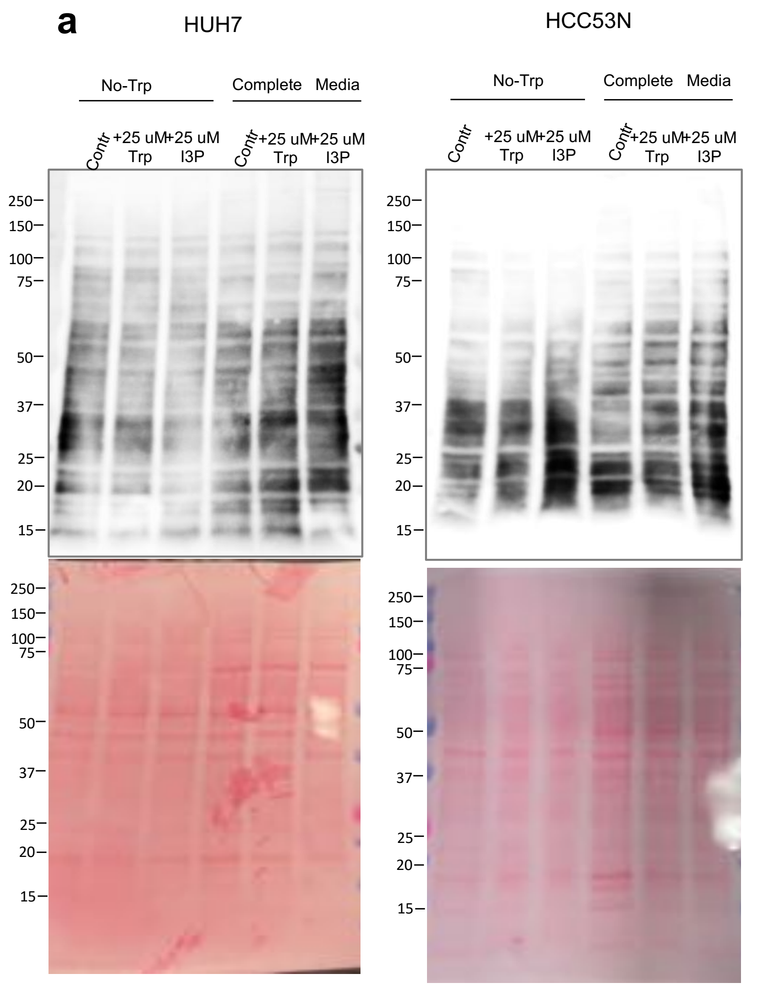
a
HUH7
No-Trp
Complete Media
Contr
+25 uM Trp
+25 uM I3P
HCC53N
250
150
100
75
50
37
25
20
15

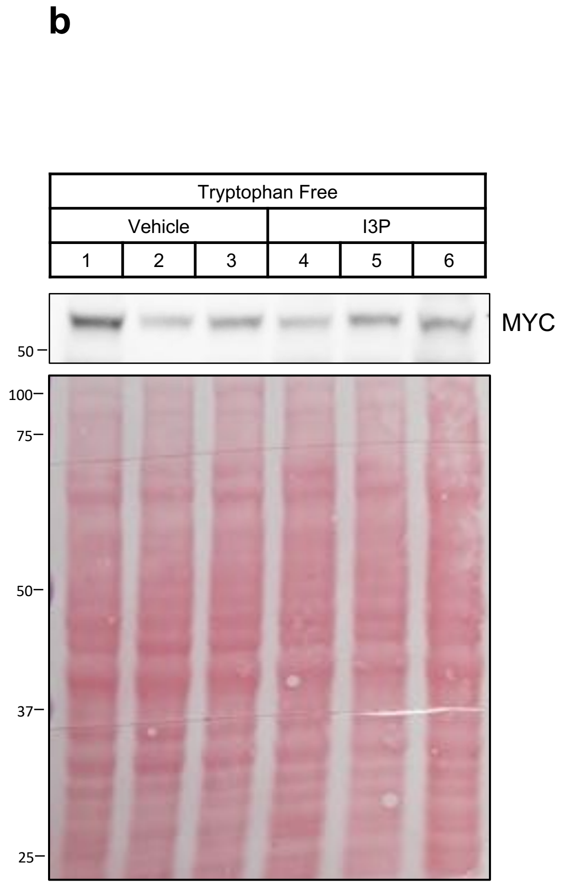
b
Tryptophan Free
Vehicle
I3P
1
2
3
4
5
6
MYC
50
100
75
37
25

Figure S9

Supplemental Fig. 9: Protein synthesis and MYC levels are not consistently altered in liver cancer cells by I3P.

(a) Puromycylation in HUH7 and HCC53N cells grown in media with (complete) or without Trp and supplemented with Trp or I3P.

(b) Expression of MYC in MYC-ON livers of mice fed a No-Trp diet for 21 days and supplemented with vehicle of I3P (see Figs 8 g-h).

Table S1: Reagents

Cell lines		
**Cell line name**	**Source**	**Catalogue number**
HUH7	JCRB	JCRB0403
SNU449	ATCC	CRL-2234
HEPG2	ATCC<br>HEPG2 cells were found in International Cell Line Authentication Committee as cross-contaminated. HEPG2 were used in this study to increase the reproducibility of our results but cells were not authenticated.	HB-8065
HCC53N	Mender, I. *et al*. Mol Cancer Ther. 2023; 22(6): 737–750[1]	N/A
THLE2	ATCC	CRL-2706

Antibodies				
**Antibody name**	**Source**	**Catalogue number**	**Dilution**	**Clone number**
MYC	Abcam	ab32072	1:10000; 1:500 (IHC)	
MAX	Santa Cruz	sc-197	1:250	
Histone H3	Cell Signaling	4499	1:1000	D1H2
SLC1A5	Abcam	ab58690	1:250	
AFMID	Sigma Aldrich	SAB1306982	1:1000	
NAMPT	Bethyl Laboratories	A700-058	1:1000	BLR058F
NMNAT	Novus	NBP1-52973	1:1000	
AHR	Enzo	BML-SA210	1:1000	
p70 S6 Kinase	Cell Signaling	2708	1:1000	49D7
Phospho-p70 S6 Kinase (Thr389)	Cell Signaling	9234	1:1000	108D2
Ki-67	Cell Signaling	12202	1:500 (IHC)	D3B5
Puromycin	Sigma Aldrich	MABE343	1:1000	12D10
β-Actin	Cell Signaling	8457	1:1000	D6A8
IL4I1	Abcam	ab222102	1:1000	EPR22070
α-Tubulin	Sigma Aldrich	T6199	1:10000	DM1A
CYP1A1	Sigma Aldrich	SAB2108545	1:1000	
NQO1	Abcam	ab80588	1:10000	EPR3309
SCIN	Sigma Aldrich	HPA020518	1:1000	
UMPS	Sigma Aldrich	HPA036178	1:1000	
AHRR	Sigma Aldrich	SAB2100078	1:1000	
Cyclin D1	Cell Signaling	2978	1:1000	92G2
Cyclin E1	Cell Signaling	20808	1:1000	D7T3U
LC3A/B	Cell Signaling	12741	1:1000	D3U4C

Atg3	Cell Signaling	3415	1:1000	
Atg5	Cell Signaling	12994	1:1000	D5F5U
Atg7	Cell Signaling	8558	1:1000	D12B11
Atg12	Cell Signaling	4180	1:1000	D88H11
Atg16L1	Cell Signaling	8089	1:1000	D6D5
Beclin-1	Cell Signaling	3495	1:1000	D40C5
ATF-4	Cell Signaling	11815	1:1000	D4B8
LC3B	Novus	NB100	1:1000	

**siRNA oligonucleotides**		
**Name**	**Source**	**Catalogue number**
Universal controls #1	Sigma mission	SIC001
siMYC, human	Sigma mission	SASI_Hs01_00222676
siSLC1A5, human	Sigma mission	SASI_Hs01_00162267
siSLC7A5, human	Sigma mission	SASI_Hs02_00335760
siMYC, mouse	Sigma mission	SASI_Mm01_00157475
siSLC1A5, mouse	Sigma mission	SASI_Mm01_00186887
siSLC7A5, mouse	Sigma mission	SASI_Mm01_00088353

**Oligonucleotides for qPCR**		
**Gene name**	**F sequence**	**R sequence**
MYC, human	GGCTCCTGGCAAAAGGTCA	CTGCGTAGTTGTGCTGATGT
SLC1A5, human	GAGCTGCTTATCCGCTTCTTC	GGGGCGTACCACATGATCC
SLC7A5, human	CCGTGAACTGCTACAGCGT	CTTCCCGATCTGGACGAAGC
MYC, mouse	CCCTATTTCATCTGCGACGAG	GAGAAGGACGTAGCGACCG
SLC1A5, mouse	CATCAACGACTCTGTTGTAGACC	CTGGATACAGGATTGCGGTATTT
SLC7A5, mouse	ATATCACGCTGCTCAACGGTG	GCCGCCTGACTTGGAGATG

Table S2: Diet composition

	Control (g/Kg)	Low Trp (g/Kg)	No-Trp (g/Kg)
L-Tryptophan	1.8	0.5	0
L-Alanine	3.5	3.5	3.5
L-Arginine HCl	12.1	12.1	12.1
L-Asparagine	6	6	6
L-Aspartic acid	3.5	3.5	3.5
L-cystine	3.5	3.5	3.5
L-Glutamic acid	40	40	40
Glycine	23.3	23.3	23.3
L-Histidine HCl	4.5	4.5	4.5
L-Isoleucine	8.2	8.2	8.2
L-Leucine	11.1	11.1	11.1
L-Lysine HCl	18	18	18
L-Methionine	8.2	8.2	8.2
L-Phenylalanine	7.5	7.5	7.5
L-Proline	3.5	3.5	3.5
L-serine	3.5	3.5	3.5
L-Threonine	8.2	8.2	8.2
L-Tyrosine	5	5	5
L-Valine	8.2	8.2	8.2
Sucrose	351.68	351.68	351.68
Corn starch	150	150	150
Maltodextrin	150	150	150
Soybean oil	80	80	80
Cellulose	30	31.3	31.8
Mineral mix	35	35	35
Calcium phosphate	8.2	8.2	8.2
Vitamin mix	13	13	13
Choline bitartrate	2.5	2.5	2.5
TBHQ, antioxidant	0.02	0.02	0.02

Table S3: Liver cancer biopsies information

PPID	Gender	Race	Ethnicity	age at collection	Histology	Primary Tumor Site	Anatomic Site	Pathological Status	Specimen Label
3_11944	Female Gender	White	Not Hispanic or Latino	74	hepatocellular carcinoma	Liver	Liver	Malignant	22552-Frozen-3
3_11944	Female Gender	White	Not Hispanic or Latino	74	hepatocellular carcinoma	Liver	Liver	Non-Malignant	22552-Frozen-2
3_13199	Male Gender	White	Not Hispanic or Latino	56	Hepatocellular carcinoma	Liver	Liver	Non-Malignant	23840-Frozen-3
3_13199	Male Gender	White	Not Hispanic or Latino	56	Hepatocellular carcinoma	Liver	Liver	Malignant	23840-Frozen-4
3_13810	Female Gender	White	Not Hispanic or Latino	79	Hepatocellular carcinoma	Liver	Liver	Malignant	24145-Frozen-4
3_13810	Female Gender	White	Not Hispanic or Latino	79	Hepatocellular carcinoma	Liver	Liver	Non-Malignant	24145-Frozen-3
3_15332	Male Gender	White	Not Hispanic or Latino	59	hepatocellular carcinoma	Liver	Liver	Malignant	24751-Frozen-5
3_15332	Male Gender	White	Not Hispanic or Latino	59	hepatocellular carcinoma	Liver	Liver	Non-Malignant	24751-Frozen-6
3_15685	Male Gender	Black or African American	Not Hispanic or Latino	58	Hepatocellular carcinoma	Liver	Liver	Malignant	25223-Frozen-3
3_15685	Male Gender	Black or African American	Not Hispanic or Latino	58	Hepatocellular carcinoma	Liver	Liver	Non-Malignant	25223-Frozen-2
3_16025	Female Gender	Black or African American	Not Hispanic or Latino	63	Hepatocellular carcinoma	Liver	Liver	Malignant	25489-Frozen-3
3_16025	Female Gender	Black or African American	Not Hispanic or Latino	63	Hepatocellular carcinoma	Liver	Liver	Non-Malignant	25489-Frozen-2
3_16736	Male Gender	Asian	Not Hispanic or Latino	83	Hepatocellular carcinoma	Liver	Liver	Non-Malignant	26124-Frozen-3
3_16736	Male Gender	Asian	Not Hispanic or Latino	83	Hepatocellular carcinoma	Liver	Liver	Malignant	26124-Frozen-4
3_17861	Male Gender	White	Not Hispanic or Latino	83	Hepatocellular carcinoma	Liver	Liver	Malignant	26546-Frozen-2
3_17861	Male Gender	White	Not Hispanic or Latino	83	Hepatocellular carcinoma	Liver	Liver	Non-Malignant	26546-Frozen-14
3_17897	Female Gender	White	Not Hispanic or Latino	54	Hepatocellular carcinoma	Liver	Liver	Non-Malignant	26591-Frozen-2
3_17897	Female Gender	White	Not Hispanic or Latino	54	Hepatocellular carcinoma	Liver	Liver	Malignant	26591-Frozen-3
3_18052	Male Gender	Black or African American	Not Hispanic or Latino	60	Hepatocellular carcinoma with neuroendocrine differentiation	Liver	Liver	Non-Malignant	26674-Frozen-4
3_18052	Male Gender	Black or African American	Not Hispanic or Latino	60	Hepatocellular carcinoma with neuroendocrine differentiation	Liver	Liver	Malignant	26674-Frozen-5

no compensation by participants
Study was approved by the UT Southwestern IRB.
Study ID: STU102010-051
Samples were provided de-identified to the researchers.
Informed consent was obtained from every patient.
Research complies with all relevant ethical regulations.

Please make sure you acknowledge the Tissue Management Shared Resource in your acknowledgements and reference the CCSG grant number.

1 Mender, I. *et al.* Activating an Adaptive Immune Response with a Telomerase-Mediated Telomere Targeting Therapeutic in Hepatocellular Carcinoma. *Mol Cancer Ther* **22**, 737-750, doi:10.1158/1535-7163.MCT-23-0039 (2023).
